# Weighted Ensemble Simulations With the Drude Polarizable Model

**DOI:** 10.1002/jcc.70264

**Published:** 2025-11-08

**Authors:** Marcelo D. Polêto, Gabriel Monteiro da Silva, Brenda M. Rubenstein, Justin A. Lemkul

**Affiliations:** ^1^ Department of Biotechnology University of São Paulo Lorena São Paulo Brazil; ^2^ Department of Biochemistry Virginia Tech Blacksburg Virginia USA; ^3^ Department of Molecular and Cellular Biology and Biochemistry Brown University Providence Rhode Island USA; ^4^ Department of Chemistry Brown University Providence Rhode Island USA; ^5^ Department of Physics Brown University Providence Rhode Island USA; ^6^ Data Science Institute Brown University Providence Rhode Island USA; ^7^ Center for Drug Discovery Virginia Tech Blacksburg Virginia USA

**Keywords:** Drude force field, enhanced sampling, molecular dynamics

## Abstract

Enhanced sampling methods have become powerful techniques to investigate complex systems and rare molecular events by facilitating greater access to configurational space. In parallel, the continuous development of polarizable force fields augments such techniques, allowing the investigation of the role of electronic polarization in transitional barriers that might be overlooked in nonpolarizable simulations. Together, improved sampling methods and polarizable force fields should accelerate computational discoveries. Here, we present an implementation of a weighted ensemble strategy for the Drude polarizable force field using the WESTPA software package in conjunction with the OpenMM simulation engine. We demonstrate its use for backbone conformational sampling in a model system (alanine dipeptide) and in studying functional sidechain rotations in an enzyme (Abl1 kinase). We further discuss possible consequences related to the inclusion of explicit electronic polarization in the model. This software implementation and validation should facilitate the use of WESTPA‐Drude strategies throughout the simulation community.

## Graphical abstract

We demonstrate the combination of the Drude polarizable force field with the WESTPA enhanced‐sampling method in the OpenMM software. Our results demonstrate the facile interaction of these two techniques and how electronic polarization impacts conformational sampling in a model alanine dipeptide system and a kinase sidechain flipping mechanism.

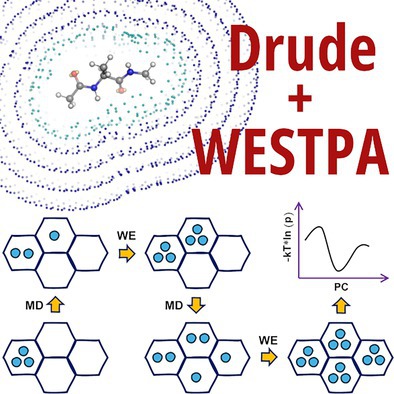

## 1 Introduction

Sampling the conformational dynamics of complex molecular systems is often the primary goal of molecular dynamics (MD) simulations, which offer atomistic details of the structural dynamics governing inter‐ and intramolecular interactions and transition events. Although the timescale of MD simulations has been steadily increasing for the past several decades [1], the observation of rare, but functionally significant molecular events is often limited by the conformational sampling of unbiased MD approaches [2]. Molecular events such as biomolecular folding or macromolecular conformational changes are usually associated with high energy barriers, requiring more elaborate strategies to achieve adequate sampling [3, 4].

In this context, weighted ensemble (WE) path sampling has shown particular success in sampling low‐probability states by weighting trajectories and binning conformational states to focus computational effort on key transitions [5, 6, 7]. The approach has found success in capturing kinetically important events such as protein folding [8, 9], ligand dissociation [10, 11, 12], large protein motions [13], and membrane permeation [14]. A key feature of the WE approach is its ability to provide statistically rigorous and reproducible transition pathways.

As free energy barriers are directly related to transition rates, it is reasonable to assume that polarizable force fields can improve sampling efficiency and produce more realistic barriers in systems for which electronic polarization modulates dynamics or stabilizes metastable transition states. In this sense, the simultaneous application of polarizable force fields with WE sampling may represent a major advancement for challenging systems in which electronic effects govern conformational changes and transition events. That is, the combination of more accurate force fields and more robust sampling approaches should yield improvements in the two main areas in which unbiased MD simulations are limited—sampling and accuracy of the physical model.

Here, we leverage the Drude polarizable force field [15] in conjunction with the Weighted Ensemble Simulation Toolkit with Parallelization and Analysis (WESTPA) package [5, 7] to describe how the inclusion of electronic polarization affects free energy barriers and transition rates.

## 2 Implementation Details

WESTPA is known for its compatibility with the most widely used MD engines in the community [7], including the OpenMM engine. The GPU acceleration provided by OpenMM has enabled extensive Drude polarizable simulations in the past [16, 17, 18, 19], making it a good candidate for extensive WE sampling.

Our approach takes advantage of native support for the Drude force field in OpenMM [20] and employs the WESTPA algorithm as an additional layer over OpenMM simulations. Here, we demonstrate the application of this OpenMM Drude implementation with a WESTPA setup on a single compute node from the Advanced Research Computing (ARC) at Virginia Tech containing 8× NVIDIA A100 GPUs. There is no requirement that the simulations performed here be carried out on this specific hardware; any multi‐GPU compute node that can run OpenMM simulations should be sufficient.

Listing 1 illustrates the directory structure assumed in our approach:

LISTING 1 **WESTPA file hierarchy.**

└─ westpa_drude/  ├── west.cfg  ├── common_files/  |   ├── toppar_drude.str  |   ├── toppar_drude/  |   |   ├── toppar_drude_master_           protein_2019h.str  |   |   ├── (other Drude force field           files)  |   ├── abl1_i1_eq_drude.psf  |   ├── abl1_i1_eq_drude.pdb  |   ├── basis.rst  |   ├── basis.pdb  |   ├── Production_drude.py  |   ├── get_dist.py  |   └── init_pcoord.py  ├── states/  |   ├── 01/  |   |   ├── basis.pdb  |   |   ├── pcoord1.dat  |   |   ├── pcoord2.dat  |   |   └── pcoord3.dat  |   └── bstates.txt  |   └── tstates.txt  ├── westpa_scripts/  |   ├── runseg.sh  |   └── get_pcoord.sh  ├── env.sh  ├── init.sh  └── submit_job.sh

The simulation directory structure begins with the main westpa_drude/ folder containing all components of our protocol. The master WESTPA configuration file (west.cfg) controls the weighted ensemble parameters, such as the number of progress coordinates (PCs), binning scheme, boundaries, propagation configuration, etc. These parameters have been summarized elsewhere [21].

The common_files/ subdirectory contains the Drude system definition: a PSF topology file, atomic coordinates in a PDB file, and a state file containing equilibrated coordinates and last frame velocities (basis.rst). Force field parameters are organized in the toppar_drude/ subfolder, which are specified in toppar_drude.str to be read when setting up the simulation context. The Production_drude.py script is called and parses input files and simulation parameters. The −state flag reads a state file either from a previous simulation interval, a parent WE simulation, or from equilibration that precedes WE simulations. The per‐iteration propagation time (τ) is defined by the −runtime flag and the iteration timestep is defined as 1 fs by the −dt flag, in accordance with the maximum allowable timestep with the Drude force field [15]. The frequency at which coordinates are saved (−savefreq) is set to the same value as τ to save only the last frame of each iteration. Finally, get_dist.py is used to calculate the PCs at the end of each iteration.

The states/ directory contains the initial and final state definitions, (bstates.txt and tstates.txt), and a subfolder containing the initial progress coordinate values.

LISTING 2 **Commands to execute WESTPA simulation in OpenMM.**

# Run the dynamics with OpenMMpython Production_drude.py −psf $WEST_SIM_ROOT/common_files/abl1_i1_eq_drude.psf \                −crd $WEST_SIM_ROOT/common_files/abl1_i1_eq_drude.pdb \                −toppar $WEST_SIM_ROOT/common_files/toppar_drude.str \                −state seg.rst \                −temp 310 \                −runtime 100 \                −savefreq 100 \                −dt 1 \                −outname seg# Calculate pcoord with MDAnalysispython3 $WEST_SIM_ROOT/common_files/get_dist.pycat dih1.dat > $WEST_DIH1_RETURNcat dih2.dat > $WEST_DIH2_RETURNpaste dist.dat dist2.dat angle1.dat > $WEST_PCOORD_RETURN

The westpa_scripts/ folder holds WESTPA execution scripts. The runseg.sh controls the simulation of each segment, and its contents are shown in Listing 2. Essentially, this script acts as a controller for the WESTPA simulation. After the simulation, progress coordinates are calculated and assigned to the $WEST_PCOORD_RETURN variable to pass to the WESTPA propagation manager.

Finally, top‐level files include configuration scripts (env.sh, init.sh) and job submission scripts for HPC environments (submit_job.sh), which should be adapted for each computational setup.

## 3 Methods

### 3.1 System Construction

First, we used alanine dipeptide as a toy model to understand the basic effects explicit electronic polarization could have on the free energy barriers associated with its conformational landscape and transition rates. To do so, we built the initial alanine dipeptide geometry and topology using the CHARMM program [22], applying the CHARMM36m force field parameters [23]. The solute was centered in a cubic box with a minimum box‐solute distance of 10 Å. The simulation box was then filled with CHARMM‐modified TIP3P water molecules [24, 25, 26]. Next, a total ionic strength of 0.15 M of KCl was added to the box to mimic cellular conditions.

The system was subsequently equilibrated for 1 ns using the OpenMM simulation engine [27] (version 7.7) while restraining the positions of non‐hydrogen solute atoms with a restraining force of 500 kJ/(mol·nm^2^). The Langevin integrator was used with a 2‐fs integration step and a temperature of 298 K, while a Monte Carlo barostat algorithm was applied to maintain the pressure at 1 bar. Electrostatic forces were calculated via the particle mesh Ewald (PME) method [28, 29] with a real‐space cutoff of 12 Å. Short‐range van der Waals forces were switched smoothly to zero from 10 to 12 Å. Bonds involving hydrogen atoms were constrained via SHAKE [30] and water molecules were constrained via SETTLE [31].

The equilibrated coordinates were converted to the Drude‐2019 polarizable force field [32] using the CHARMM program. The TIP3P representation of water was converted to the polarizable SWM4‐NDP model [33]. The energy of the system was minimized by allowing the positions of the Drude oscillators to relax. Minimization was performed via 1000 steps of the steepest descent algorithm, followed by an additional 500 steps of the adopted‐basis Newton–Raphson algorithm. The system was equilibrated in OpenMM [20, 27] for 1 ns using a Langevin integrator with a 1‐fs integration step. The temperature was controlled by a dual Langevin thermostat scheme coupling the Drude oscillators to a relative thermostat at 1 K while real atoms were coupled to a thermostat at 300 K. The pressure was kept at 1 bar via a Monte Carlo algorithm. The non‐bonded treatment used for the Drude system employed the same parameters as those used for the additive systems, with the exception of a switching function applied to van der Waals potentials from 10 to 12 Å, as specified by the force field convention [15]. A polarization catastrophe was avoided by applying the Drude hard wall constraint [34], allowing a maximum Drude‐atom bond length of 0.2 Å.

To build the Abl1 kinase system, we opted to start with the “DFG‐in” (active) conformation (PDB:6XR6) [35]. The protein topology was generated using the CHARMM36m force field in the CHARMM program. Asp381 was protonated following previous literature [36]. The protein was then centered in a simulation box with a minimum distance of 10 Å from the closest protein atom to the box edge. The system was filled with CHARMM‐modified TIP3P water molecules and the net charge on the system was neutralized by adding 0.15 M of KCl, including counterions. The Abl1 system was then minimized and equilibrated with CHARMM36m and converted to the Drude polarizable model using the same procedures described above for the alanine dipeptide. The equilibrated coordinates for the additive and polarizable counterparts of both systems were used in the subsequent WE simulations.

### 3.2 WESTPA Parameters

For the alanine dipeptide systems, we chose the backbone torsions ϕ and ψ as progress coordinates to describe the conformational sampling of the peptide since most of its conformational ensemble can be interpreted based on these two dimensions. For each of these progress coordinates, we defined a single bin spanning from −180° to 180°, and divided it into 10 dynamic bins using the minimal, adaptive binning (MAB) scheme to discretize the PC space and direct resources to potential bottlenecks [37]. The MAB scheme further accelerates sampling by dynamically adjusting bins based on PC evolution to enhance the sampling in bottleneck regions of PC space while maintaining a near‐constant computational load. We then set the number of walkers per bin to 10, totaling a maximum of 1210 parallel walkers when all bins are occupied (11 bins per coordinate, 10 walkers per bin). WE simulations ran for 100 iterations, with a τ of 100 ps. Each WE run was performed in three replicates.

**FIGURE 1:**
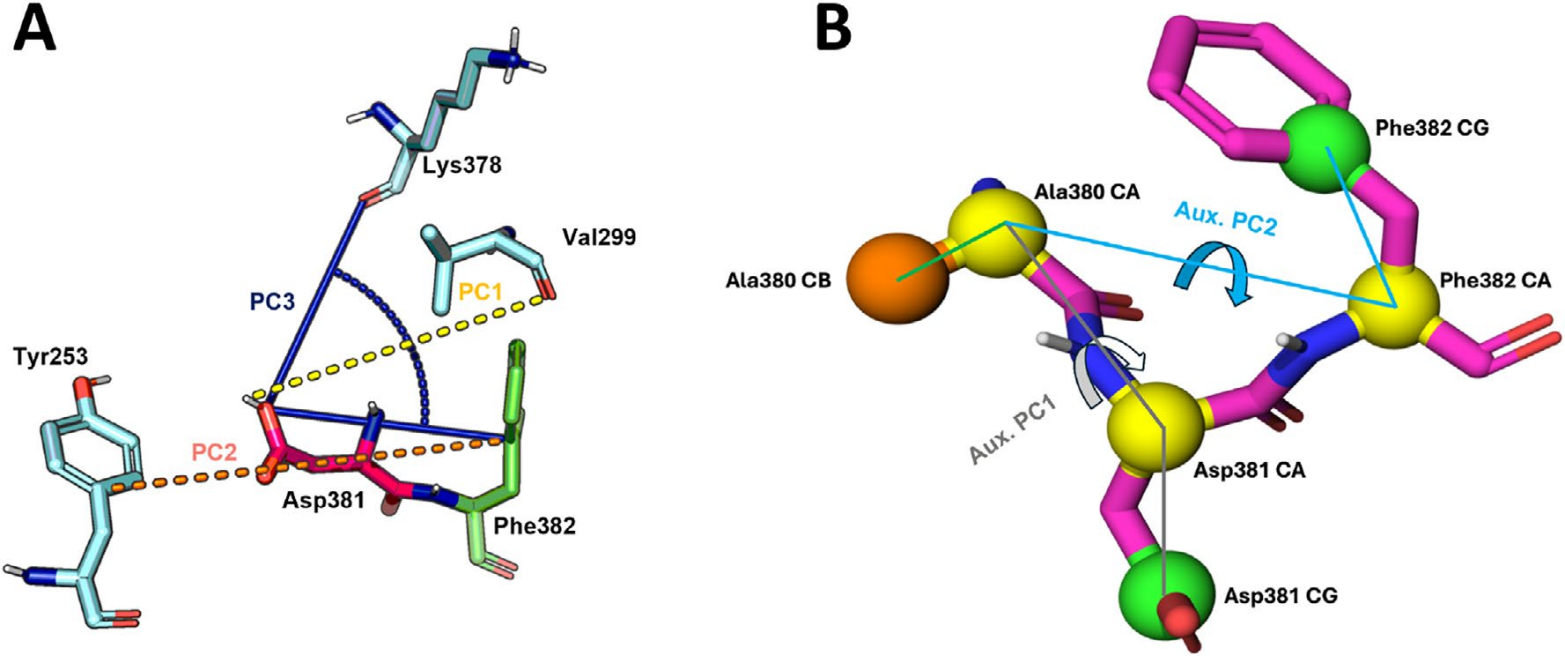
Illustration of the progress coordinates used in the Abl1 system. (A) Progress coordinates used to enhance the sampling towards the target state (DFG out). (B) Auxiliary progress coordinates used for analysis and visualization.

In the Abl1 system, to enhance the sampling of the simulations starting from the DFG in state towards the target DFG out state, we used three previously validated progress coordinates (Figure 1A). For PC1, we used the distance between the polar hydrogen of protonated Asp381 and the backbone oxygen of Val299 as PC1, as DFG flip studies using long simulations have shown that a transient hydrogen interaction between these two atoms might facilitate a DFG flip event [36, 38]. For PC2, we used the distance between the γ carbons of Phe382 and Tyr253, which has been demonstrated to enhance the sampling of Phe382 flipping [38], and for PC3, we used the angle formed between the γ carbon of Phe382, the protonated side chain oxygen of Asp381, and the backbone oxygen of Lys381 as PC3 based on prior literature using an equivalent PC to simulate the DFG flip in Aurora Kinase B with metadynamics [39].

To unambiguously track the evolution of the DFG flip in Abl1 (i.e., the conversion from “DFG‐in” to “DFG‐out”) and to facilitate visualization, we used pseudodihedrals previously defined in the Abl1 DFG flip simulation literature as auxiliary progress coordinates (Aux. PCs) [40], as shown in Figure 1B. Importantly, these observables are not used to direct the simulations and thus do not enhance the sampling. Instead, they are used for facilitating downstream analysis and visualization. First, we defined Aux. PC1 as the pseudodihedral formed by $\mathrm{Ala}380\left(C\beta \right)–\mathrm{Ala}380\left(C\alpha \right)–\mathrm{Asp}381\left(C\alpha \right)–\mathrm{Asp}381\left(C\gamma \right)$. As evidenced by the choice of atoms forming the pseudodihedral, Aux. PC1 tracks the flipping of the Asp381 sidechain relative to a well‐defined position (the backbone of Ala380). Similarly, Aux. PC2 tracks the flipping of Phe382 through the pseudodihedral formed by $\mathrm{Ala}380\left(C\beta \right)–\mathrm{Ala}380\left(C\alpha \right)–\mathrm{Phe}382\left(C\alpha \right)–\mathrm{Phe}382\left(C\gamma \right)$.

Having chosen our set of PCs, we ran WE simulations of wild‐type Abl1 starting in the DFG‐in conformation with the DFG‐out conformation as a target state, using the apo Abl1 DFG‐in and DFG‐out structures as references (PDB:6XR6 and 6XR7, respectively) [35]. As in the alanine dipeptide system, we used the MAB scheme to optimize the use of computational resources. WE simulations ran for 125 iterations, with a τ of 100 ps. Each WE run was performed in three replicates. For both the alanine dipeptide and Abl1 systems, the MD simulations were conducted using the same simulation settings as used for equilibration, with the Abl1 system running at a temperature of 310 K due to the anticipated high free energy barrier of the DFG flip.

## 4 Results and Discussion

### 4.1 Alanine Dipeptide as a Model System

**FIGURE 2:**
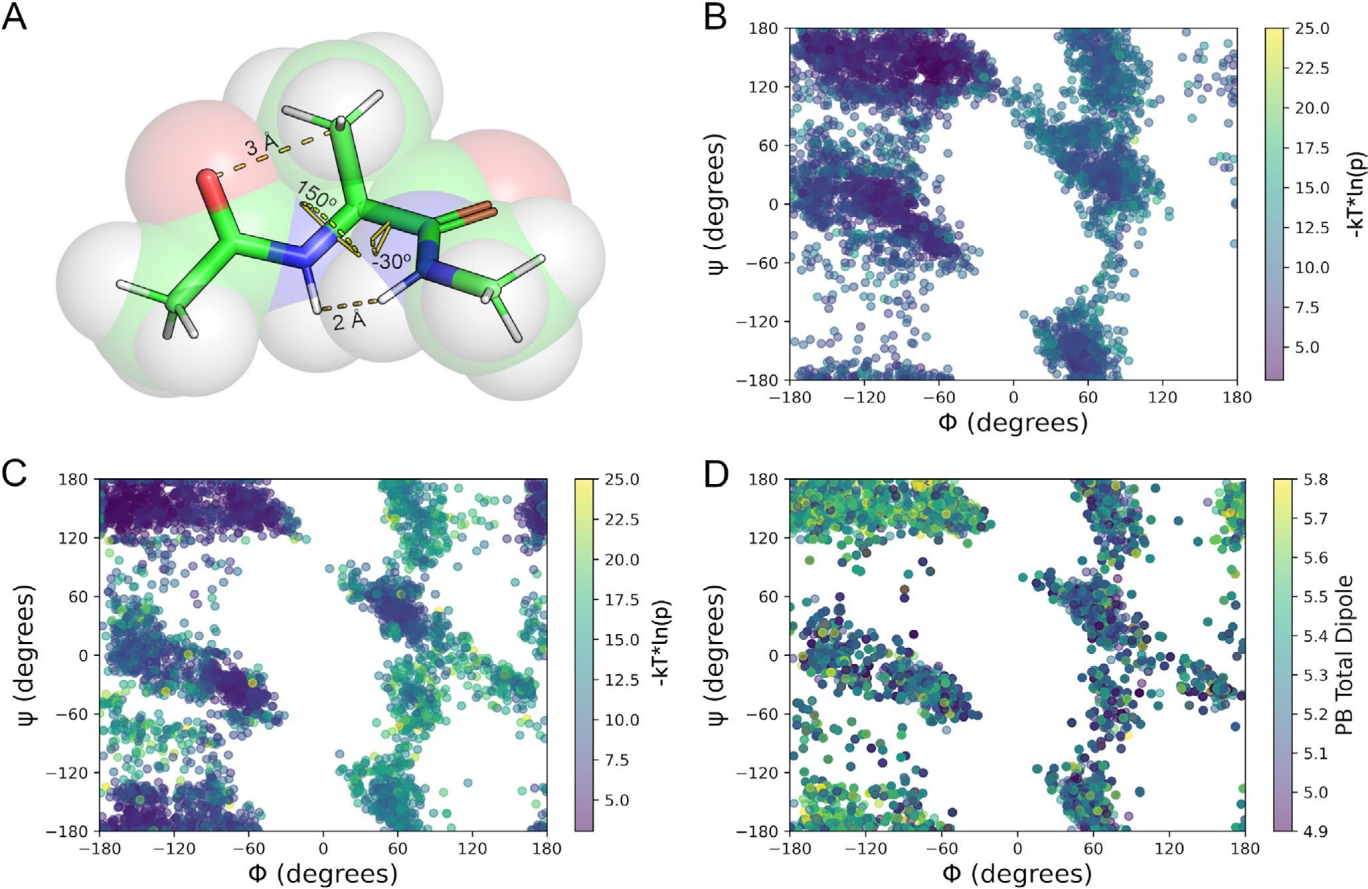
(A) Alanine dipeptide conformation with $\phi =150^{{}^{\circ}}$ and $\psi =-30^{{}^{\circ}}$, highlighting the electrostatic clash between peptide bond protons and between the carbonyl oxygen and methyl side chain group. (B, C) Probability profiles for the alanine dipeptide systems using the CHARMM36m and Drude force fields, respectively. (D) Peptide‐bond dipole moments (in Debye) as a function of the ϕ and ψ PCs in the Drude polarizable simulation.

The evolution of conformational sampling through a given PC is directly tied to the magnitude of the free energy barriers separating the states. Overcoming these barriers often requires resolving electronically unfavorable interactions, either within the solute or between the solute and the solvent. For instance, some combinations of the ϕ and ψ angles of alanine dipeptide can force distinct chemical groups into steric clashes due to overlapping electron clouds. For example, as shown in Figure 2A, a $\phi =150^{{}^{\circ}}$ angle induces a van der Waals clash between the carbonyl oxygen in the backbone and the methyl group of the side chain. However, when combined with $\psi =-30^{{}^{\circ}}$, this conformation leads to an additional electrostatic clash between the two peptide bond amide protons that creates a localized high‐energy state that may hinder conformational transitions, directly shaping the free energy landscape.

In additive force fields, these repulsions are modeled using Coulomb's Law with atom‐centered fixed charges, which cannot account for the redistribution of electron density that occurs when atoms approach each other closely. Thus, the repulsion calculated from such models would be overestimated because the model cannot “sense” the proximity‐driven polarization that partially mitigates the interaction in reality.

In contrast, polarizable models such as the Drude force field incorporate electronic polarization by allowing charge distributions to dynamically adjust in response to the local electric field. For repulsive cases, the Drude model redistributes electron density to reduce the net repulsion, mimicking the natural electronic shielding and softening the steric strain. This dynamic adaptation results in free energy profiles with lower barriers compared to additive models, more closely replicating the electronic plasticity seen in realistic biomolecules.

The conformations commonly adopted by alanine dipeptide are shown in Figure S1. As shown in Figure 2B,C, both the CHARMM36m and Drude models sampled the C5, PPII, $\alpha_{R}$, $\alpha_{L}$, and ${\text{C7}}_{\mathrm{ax}}$ regions, in agreement with previous reports [41, 42]. Here, the ${\text{C7}}_{\mathrm{ax}}$ conformation was less populated by the Drude‐2019 model than by CHARMM36m. In fact, conformations in which $\phi \approx 60^{{}^{\circ}}$ for various ψ values yielded larger free energy values than those from the CHARMM36m simulations, with the exception of the $\alpha_{L}$ regions. Interestingly, the Drude simulations also sampled regions with electrostatic clashes, such as the one shown in Figure 2A, with $\phi \approx 150^{{}^{\circ}}$ and $\psi \approx -30^{{}^{\circ}}$. This region of (ϕ, ψ) space was not sampled in the CHARMM36m simulations, suggesting that the inclusion of electronic polarization may soften this interaction that would otherwise be strongly repulsive with a nonpolarizable force field.

We also investigated the relationship between backbone polarization and the conformational space of the alanine dipeptide by calculating the total peptide bond dipole moments ($\mu_{\mathrm{PB}}$) of our Drude simulations. As shown in Figure 2D, the values of $\mu_{\mathrm{PB}}$ ranged from 4.6 D to 5.8 D, with larger values in the C5 and PPII regions. In contrast, the $\alpha_{R}$ and $\alpha_{L}$ regions produced smaller dipole moments, in line with reports of this phenomenon in globular proteins [42].

**FIGURE 3:**
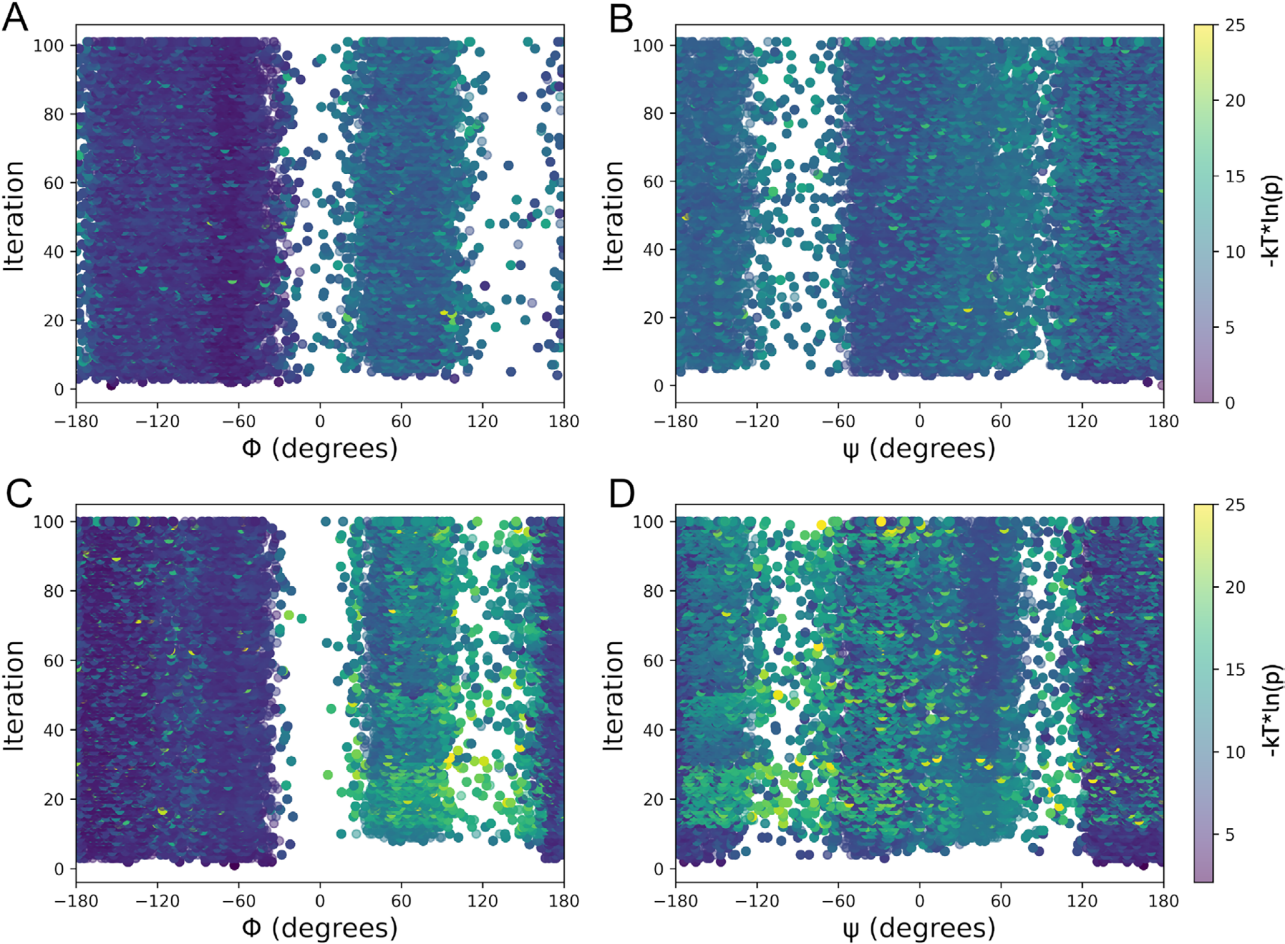
The evolution of ϕ and ψ for CHARMM36m (A and B, respectively) and Drude (C and D, respectively).

As shown in Figure 3A–D, both additive and polarizable simulations extensively sampled ϕ and ψ dihedral space within the first few WESTPA iterations. However, the Drude model sampled high free energy states at $90^{{}^{\circ}}<\phi <165^{{}^{\circ}}$ and $-120^{{}^{\circ}}<\psi <-60^{{}^{\circ}}$, two regions rarely sampled in the additive simulations (Figure 3C). These results demonstrate how electronic polarization modulates free energy barriers and impacts conformational sampling.

### 4.2 Abl1 Kinase

Protein tyrosine kinases play a crucial role in cellular signaling pathways by transferring the γ‐phosphate of an ATP to tyrosine residues in substrate proteins. In doing so, these enzymes regulate cell proliferation, metabolism, and differentiation [43]. While Abl1 kinase is known to regulate various cellular processes, the abnormal translocation of its gene and fusion of the BCR and Abl1 genes lead to chronic myeloid leukemia [43, 44].

**FIGURE 4:**
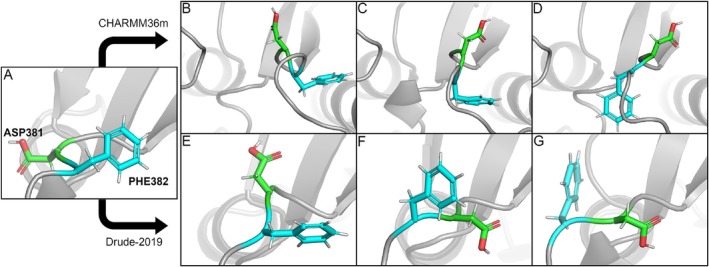
DFG flip for Abl1. (A) Initial DFG‐in state used for both the CHARMM36m and Drude simulations. The pathway towards a DFG‐out state is shown in (B–D) from the CHARMM36 simulation, while an alternative pathway is shown in (E–G) from the Drude simulation.

The DFG motif (Asp381 – Phe382 – Gly383) present in the activation loop (A loop) is known to regulate Abl1 activity. In the active state (DFG‐in, Figure 4A), the Asp381 residue is oriented towards the active site and coordinates a catalytic Mg^2+^ ion. The inactive state (DFG‐out) is characterized by an inversion in the side chain orientations of the DFG motif, in which Phe382 shifts inward to the active site while Asp381 moves out. This DFG‐flip mechanism has been studied comprehensively, and it is considered a form of Abl1 self‐inhibition by intrinsic dynamics [38, 39, 40, 45]. Previous studies have shown that the propensity to undergo DFG flips is linked to the protonation state of Asp381, as protonation greatly increases the DFG flip probability [36].

Here, we employed WESTPA simulations to investigate the role of electronic polarization in the DFG flip mechanism of Abl1 kinase. As shown in Figure 4, the CHARMM36m and Drude systems sampled distinct conformational pathways for the DFG flip. In both systems, Aux. PC1 (which tracks the flip of Asp381) followed a similar trajectory, transitioning from ≈220° to ≈3°, forming a transient conformation (known as DFG inter, as both Asp381 and Phe382 point away from the active site) that has been observed in other structural studies of kinases [38, 39]. However, the two systems exhibited divergent behavior along Aux. PC2 (which tracks the flip of Phe382). For CHARMM36m, Aux. PC2 initially resided at ≈50° (Figure 4B), decreased towards 0° (Figure 4C), crossed over to ≈330°, and eventually stabilized at ≈220° (Figure 4D). In contrast, the Drude system moved along a different pathway, with PC2 evolving from ≈50° (Figure 4E) to ≈100° (Figure 4F), before stabilizing at ≈200° (Figure 4G). These differences suggest that the inclusion of explicit electronic polarization could enable the exploration of multiple DFG‐flip pathways that have not yet been explored without the use of biasing forces, or motivate a new hypothesis for an alternate pathway that should be explored.

In addition, the free energy landscapes generated by the two systems showed distinct differences, which provide evidence that this difference in pathways is not an anomaly from a single trajectory pair, but rather a statistically meaningful difference in pathway preference replicated across an ensemble of parallel simulations. Although the DFG‐in state is estimated to be the global free energy minimum for both systems, all intermediate states leading to DFG‐out corresponded to free energy values exceeding 30 kcal/mol for the CHARMM36m system. In contrast, the Drude system predicted a metastable intermediate state, characterized by a local free energy minimum in the region (0° < Aux. PC1 < 70°, 0° < Aux. PC2 < 70°), which subsequently transitioned to the DFG‐out state.

**FIGURE 5:**
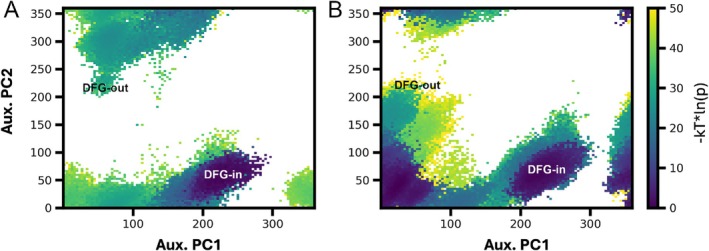
Two‐dimensional projections of Aux. PC1 and Aux. PC2 from the entire WE simulation dataset for (A) CHARMM36m and (B) Drude systems.

Another important difference can be observed in the phase space sampling. As shown in Figure 5A,B, the CHARMM36m system explored less of the (Aux. PC1, Aux. PC2) phase space than the Drude system. In fact, the Drude simulations were able to explore high free energy states (≈50 kcal/mol) much more frequently. Together, these findings suggest that the inclusion of explicit electronic polarization can facilitate phase space sampling by resolving electronic clashes more readily.

**FIGURE 6:**
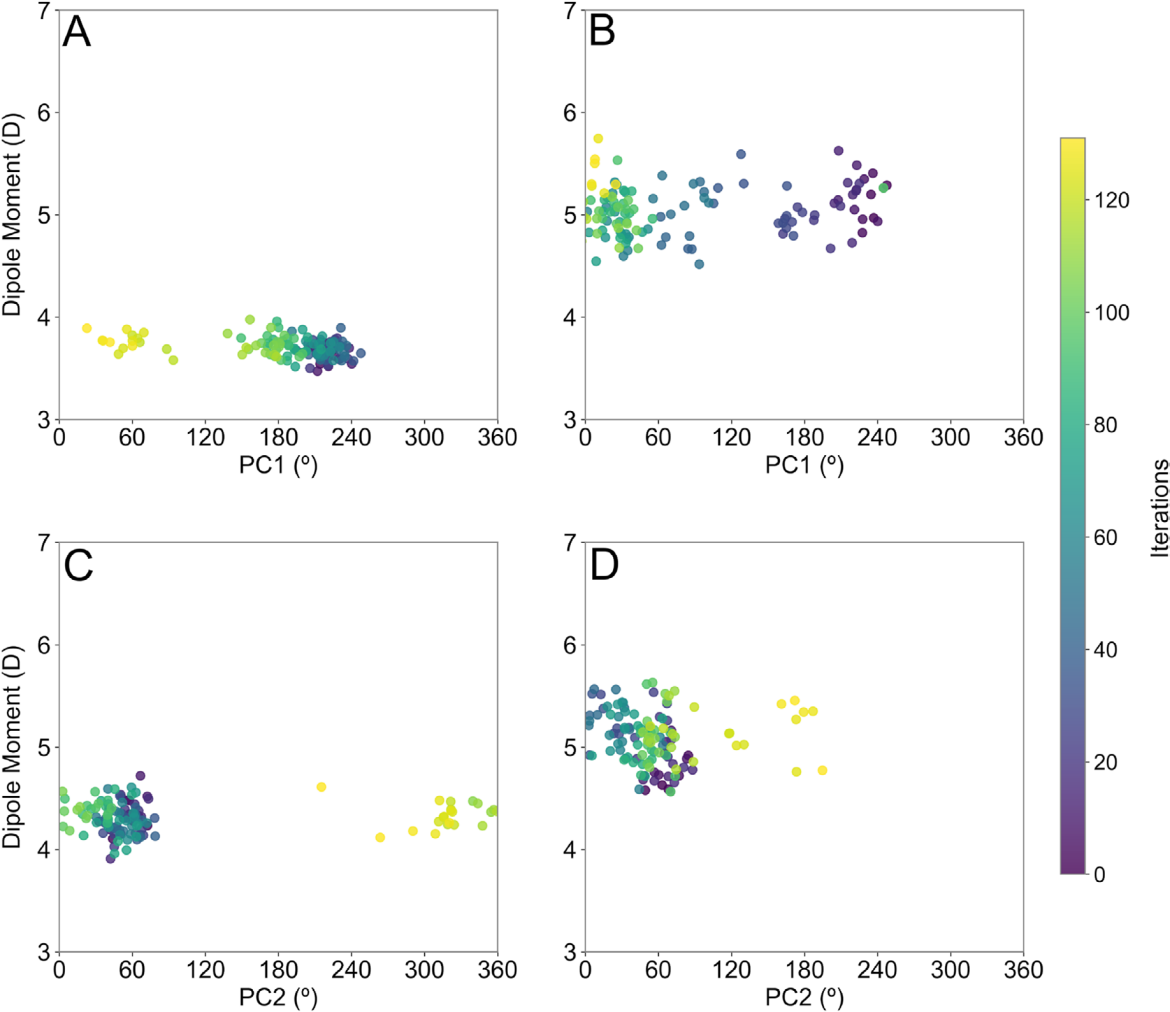
Time evolution of the peptide bond dipole moment ($\mu_{\mathrm{PB}}$, in Debye) as a function of Aux. PC1 and Aux. PC2. (A) Asp381 for the CHARMM36m system. (B) Asp381 for the Drude system. (C) Phe382 for the CHARMM36m system. (D) Phe382 for the Drude system.

To investigate this hypothesis (i.e., the role of electronic polarization in the pathway transitions), we collated frames from all of the WE walkers that traversed the lowest‐energy transition pathway in each system. To do so, we used the *W_TRACE* feature in WESTPA2 [7] to trace the entire file system history of walkers that reached the target state (DFG‐out) and collated their trajectory data using MDTraj [46] to obtain two pseudotrajectories that represent the DFG‐flip transition pathway for each system. We then calculated the $\mu_{\mathrm{PB}}$, the peptide bond dipole moment, of residues Asp381 and Phe382 as a function of the pathway. As shown in Figure 6A,B, the change in Aux. PC1 is followed by a change of the ϕ dihedral angle of Asp381, which impacts the magnitude of its peptide bond dipole moment. For CHARMM36m, $\mu_{\mathrm{PB}}$ of Asp381 is close to 3.6 D in the DFG‐in state and slightly elevated (≈3.8 D) in the DFG‐out state. For Drude, $\mu_{\mathrm{PB}}$ of Asp381 presents much more variation along the pathway, starting closer to ≈5 D in the DFG‐in state and reaching values of ≈5.6 D in the DFG‐out state. We also calculated $\mu_{\mathrm{PB}}$ for Phe382 and its relationship with the DFG‐flip as a function of Aux. PC2 (Figure 6C,D). While $\mu_{\mathrm{PB}}$ was ≈4.4 D for both the DFG‐in and DFG‐out states in our CHARMM36m system (Figure 6C), the Drude systems showed much more electronic variability, with $\mu_{\mathrm{PB}}$ varying from ≈4.6 D in the DFG‐in state, reaching values of 5.6 D along the pathway, and finally reaching ≈5.2 D in the DFG‐out state.

We also calculated the sidechain dipole moment ($\mu_{\mathrm{SC}}$) of residues Asp381 and Phe382 as a function of the pathway. As shown in Figure S2A, $\mu_{\mathrm{SC}}$ of Asp381 in our CHARMM36m simulations was close to 1.5 D in the DFG‐in state, reaching 2 D in the DFG‐out state. For Drude simulations, $\mu_{\mathrm{SC}}$ of Asp381 was ≈3 D for the DFG‐in and DFG‐out states but sampled a greater range of values along the pathway, ranging from ≈2–4 D (Figure S2B). Similarly, $\mu_{\mathrm{SC}}$ of Phe382 was close to 0.5 D in both the DFG‐in and DFG‐out states in our CHARMM36m simulations (Figure S2C), while $\mu_{\mathrm{SC}}$ of Phe382 in Drude adopted a wider range of values in the DFG‐in state (from ≈0.5 D to ≈2 D), and $\mu_{\mathrm{SC}}$ closer to 1.5 D in the DFG‐out state (Figure S2D). Together, the change in $\mu_{\mathrm{PB}}$ as a function of the DFG‐flip discussed above, the broader distribution of $\mu_{\mathrm{SC}}$ values sampled along the pathway, and the accompanying wider region of configuration space sampled suggest that the inclusion of explicit electronic polarization may enable conformational transitions by allowing for response to the microenvironment due to the inherent plasticity of the electrostatic model.

Our results demonstrate a relationship between the conformational dynamics and the electronic polarization of residues involved in the DFG‐flip mechanism, impacting the transition pathways sampled. Although our CHARMM36m model reproduced a previously reported flip pathway [38], the polarizable Drude simulations revealed an alternative pathway with different energetics. A complete characterization of these polarization‐dependent effects, including potentially novel DFG‐flip pathways, would require additional replicates and a convergence assessment, which is beyond the scope of our report. Here, we aimed to demonstrate possible benefits of using a polarizable model such as Drude in WE simulations.

## 5 Conclusions

Here, we have described the implementation of a WE approach using the Drude polarizable force field and discussed the behavior of the resulting polarizable WESTPA simulations, including the impact of different electrostatic treatments on free energy barriers, sampling of alternative transition pathways along common PCs, and changes in dipole moment as a function of progress coordinates. We demonstrated in an alanine dipeptide model how the inclusion of electronic polarization enhances the conformational sampling due to a more physical treatment of electrostatic interactions, accessing states that would be considered high energy to additive models. We also employed our approach on a larger, more biologically relevant system, the DFG‐flip mechanism in Abl1 kinase. Our Drude simulations sampled a DFG‐flip pathway that was accompanied by changes in the peptide‐bond and sidechain dipole moments of Asp381 and Phe382, creating a metastable state between the DFG‐in and DFG‐out states that was not observed using the CHARMM36m model. Further investigation of this DFG‐flip mechanism is needed to assess the convergence and relevance of the observed pathways. Here, our goal was to establish a WESTPA‐Drude implementation that is easy to deploy and illustrates its application to well‐known biomolecular examples.

## Conflicts of Interest

The authors declare no conflicts of interest.

## Supporting information


**Figure S1:** Conformations commonly adopted by alanine dipeptide.
**Figure S2:** Time evolution of sidechain dipole moment (μSC, in Debye) as a function of PC1 and PC2. Asp381 for (A) CHARMM36m and (B) Drude simulations. Phe382 for (C) CHARMM36m and (D) Drude simulations.

## Data Availability

The data that support the findings of this study are openly available in GitHub at https://github.com/Lemkul‐Lab/westpa_drude.
