# Supplementary material for: Weighted Ensemble Simulations With the Drude Polarizable Model
Source: J Comput Chem. 2025 Nov 8;46(30):e70264. doi: 10.1002/jcc.70264 (PMC12595399; doi:10.1002/jcc.70264)
Supplement: Supplementary file 1 — Figure S1: Conformations commonly adopted by alanine dipeptide. Figure S2: Time evolution of sidechain dipole moment (μSC, in Debye) as a function of PC1 and PC2. Asp381 for (A) CHARMM36m and (B) Drude simulations. Phe382 for (C) CHARMM36m and (D) Drude simulations. [file JCC-46-0-s001.pdf]

SOFTWARENOTE

SUPPORTING INFORMATION

Weighted ensemble simulations with the Drude polarizable model

Marcelo D. Polêto[1,2*] | Gabriel Monteiro da Silva[3*] | Brenda M. Rubenstein[4,5,6] | Justin A. Lemkul[2,7]

*Equally contributing authors.

SUPPORTING FIGURES

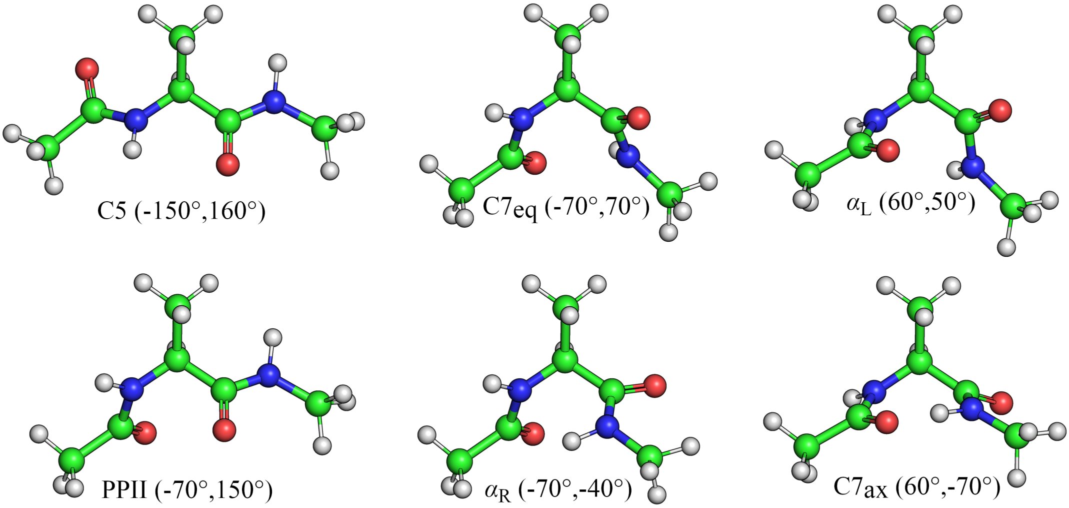

FIGURE S1 Conformations commonly adopted by alanine dipeptide.

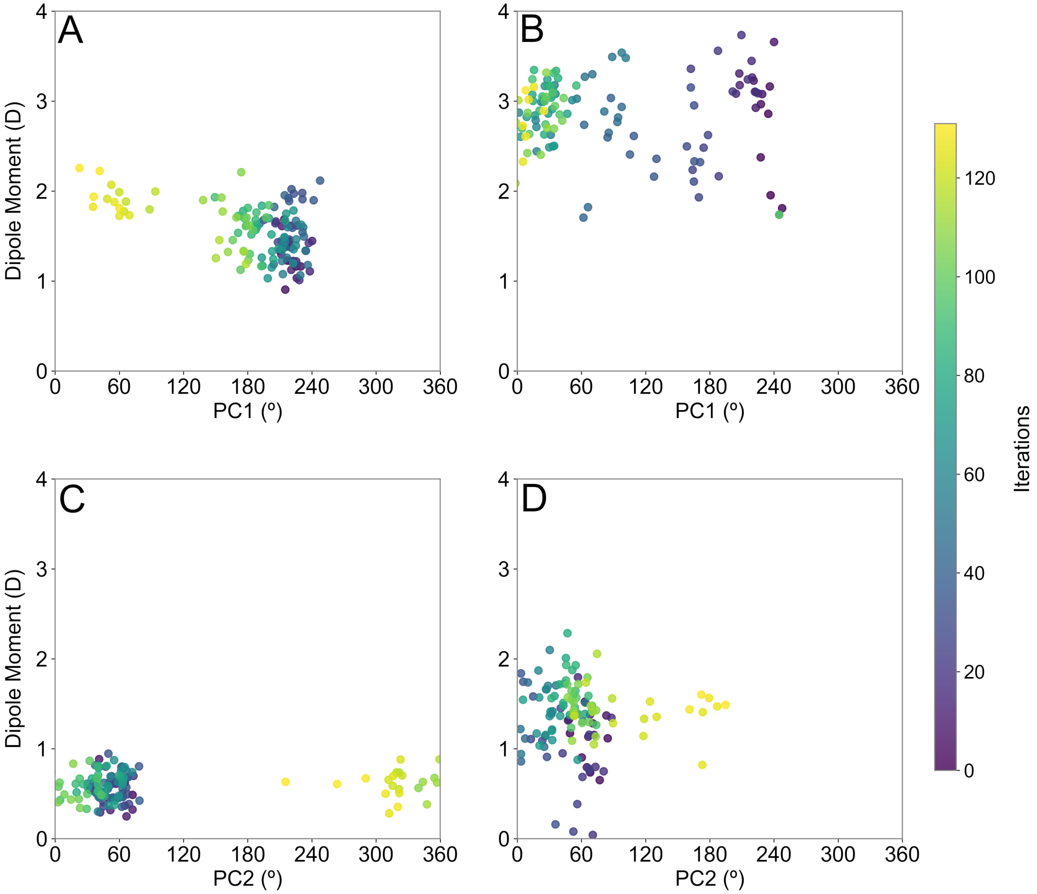

FIGURE S2 Time evolution of sidechain dipole moment (μ_{SC}, in Debye) as a function of PC1 and PC2. Asp381 for (A) CHARMM36m and (B) Drude simulations. Phe382 for (C) CHARMM36m and (D) Drude simulations.
